# Collision Tumor of the Thyroid Gland: Primary Squamous Cell and Papillary Thyroid Carcinoma

**DOI:** 10.5402/2011/582374

**Published:** 2011-05-31

**Authors:** Meir Warman, Noga Lipschitz, Sergey Ikher, Doron Halperin

**Affiliations:** ^1^Department of Otolaryngology, Head and Neck Surgery, Kaplan Medical Center, P.O.B. 1, Rehovot 76100, Israel; ^2^Hebrew University Hadassah Medical School, Ein Kerem, P.O.B. 1200, Jerusalem 91120, Israel; ^3^Sackler Faculty of Medicine, Tel-Aviv University, Ramat Aviv, P.O.B. 39040, Tel-Aviv 69978, Israel; ^4^Department of Pathology, Kaplan Medical Center, P.O.B. 1, Rehovot 76100, Israel

## Abstract

*Introduction*. Collision tumor of the thyroid gland is defined when independent and histologically distinct tumors coexist within the gland. The presence of both papillary and squamous cell carcinoma in the thyroid gland is unusual. Suggested etiologies include embryonic remanents of squamous epithelium, chronic inflammation, or thyroid malignancies promoting squamous metaplasia. *Case Presentation*. An elderly patient presented with a rapid enlargement of a long-standing right thyroid nodule. The tumor was locally invasive and unresectable. Pathology revealed the diagnosis of papillary and squamous cell carcinoma of the thyroid gland. Possible primary sites for squamous cell carcinoma in upper aerodigestive tract were excluded. The patient outcome was fatal although palliative chemoradiotherapy. *Discussion*. Collision tumor of papillary and squamous cell carcinoma of the thyroid gland is a rare entity that may imply bad prognosis, as to the presence of the squamous portion. The best treatment includes resection of the tumor; unfortunately it is not possible in most cases.

## 1. Case Report

An 84-year-old woman known to have a long-standing right thyroid nodule presented with a rapidly enlarging right neck mass. She reported dysphagia and regional pain radiating to the chest and ipsilateral ear. Physical examination demonstrated solid and fixed right thyroid mass with normal vocal cord motility and no cervical lymphadenopathy. Ultrasonography revealed a 5 × 3 × 3 cm right thyroid mass with cystic and solid components and coarse calcification foci. Fine needle aspiration cytology showed abundant inflammatory cells and foamy macrophages. At operation, a locally invasive tumor was found, involving the neck skin and infiltrating the larynx and tracheal cartilages, vascular compartments, and prevertebral fascia, therefore being unresectable. Frozen section biopsies ruled out anaplastic carcinoma, and partial thyroidectomy without tracheostomy was performed. Postoperatively computed tomography with contrast material demonstrated infiltrative lesion invading the laryngeal cartilages and vascular compartment with no evidence of other primary head and neck carcinoma. 

**Figure 1:**
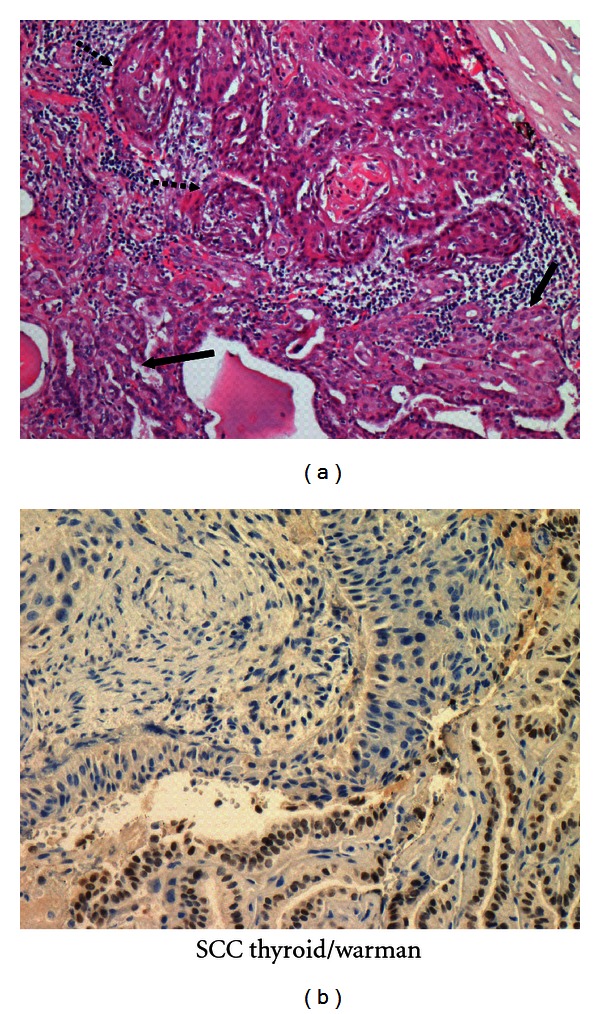
(a) Histological examination shows two distinct areas, one with islands of squamous cells in various stages of differentiation, intercellular bridges, and small keratin pearls (black dotted arrows) and the other with papillae lined by cuboidal cells with overlapping nuclei and finely dispersed optically clear chromatin (black thick arrows), (Hematoxyllin and Eosin X200). (b) The tumor shows positive nuclear stain for TTF-1 in papillary area.

Histological examination (Figure 1(a)) showed two distinct areas, one with islands of squamous cells in various stages of differentiation, intercellular bridges, and small keratin pearls and another with papillae lined by cuboidal cells with overlapping nuclei and finely dispersed clear chromatin. Between these two areas, an extensive lymphoplasmacytic infiltrate was noted. Immunohistochemical stains demonstrated positive nuclear stain for TTF-1 of the papillary carcinoma (Figure 1(b)) and positive cytoplasmic CK-5 staining with of the squamous area.

The coexistence of papillary thyroid carcinoma and squamous cell carcinoma in separate areas of the same specimen was consistent with the diagnosis of collision tumor of the thyroid gland.

The patient received external beam radiation therapy (70 Gy). Shortly after, she was diagnosed with metastatic axillary lymph node involvement and died within a few months. This paper was approved by the Kaplan medical center ethical committee.

## 2. Discussion

The term “collision tumor” refers to coexistence of independent tumors that are histologically distinct. While multicentricity of papillary thyroid carcinoma is not uncommon, the presence of two histologically separate primary neoplasms within the thyroid gland is unusual [1]. 

Papillary thyroid carcinoma represents the most common thyroid malignancy, whereas primary squamous cell carcinoma (SCC) of the thyroid gland is a rare clinical entity, which accounts for less than 1% of all thyroid malignancies. 

The pathogenesis of primary SCC of the thyroid remains unclear, since the gland does not normally contain squamous epithelium. Possible exceptions, in which squamous cells can be found in the thyroid, include embryonic remnants, inflammatory processes, and neoplasms [2, 3]. Chronic inflammation, present in Hashimoto thyroiditis, nodular goiter, or thyroid malignancies, can promote squamous metaplasia of the follicular epithelium, which might undergo malignant transformation into SCC [3]. Primary SCC of the thyroid, whether pure SCC or SCC arising in association with papillary carcinoma, is more common in women and typically affects patients in their 5th and 6th decades [2]. However, patients' ages vary in previous reports, ranging from 30 to 89 years [4]. Papillary thyroid carcinoma is a tumor with an indolent nature that is more common in women and typically affects patients in their 3rd and 4th decades. One could assume that the transitional process in which squamous metaplasia develops in papillary carcinoma and undergoes malignant transformation to SCC progresses through many years, explaining the similar female susceptibility yet different age at presentation between these two entities. In our patient specimen however, we did not find squamous metaplasia which may suggest de novo appearance of primary SCC from follicular epithelial cells.

SCC of the thyroid typically presents as a rapidly enlarging neck mass, often associated with pain, dysphagia, dyspnea, and hoarseness [2]. Local invasion to adjacent structures and early metastatic spread are common. Diagnostic workup should be preformed to rule out extrathyroid primary SCC as an origin for the thyroid SCC. In our patient, clinical examination and a CT scan did not reveal another possible site as the origin for the SCC. The treatment of SCC of the thyroid is not well defined and usually involves multiple modalities. Surgical resection when feasible can provide the potential for cure. Palliative surgery is often recommended to treat airway compromise [2]. In addition, primary SCC of the thyroid is considered resistant to radiotherapy and chemotherapy, yet adjuvant postoperative radiotherapy is generally recommended and has been associated with better survival rates [5].
